# The Story behind the Mask: A Narrative Review on Hypomimia in Parkinson’s Disease

**DOI:** 10.3390/brainsci14010109

**Published:** 2024-01-22

**Authors:** Edoardo Bianchini, Domiziana Rinaldi, Marika Alborghetti, Marta Simonelli, Flavia D’Audino, Camilla Onelli, Elena Pegolo, Francesco E. Pontieri

**Affiliations:** 1 Department of Neuroscience, Mental Health and Sensory Organs (NESMOS), Sapienza University of Rome, 00189 Rome, Italy; edoardo.bianchini@uniroma1.it (E.B.); domiziana.rinaldi@gmail.com (D.R.); marika.alborghetti@gmail.com (M.A.); marta.simonelli@uniroma1.it (M.S.); 2 AGEIS, Université Grenoble Alpes, 38000 Grenoble, France; 3 Sant’Andrea University Hospital, 00189 Rome, Italy; flavia.daudino@gmail.com; 4 Ospedale dei Castelli, ASL Rome 6, 00040 Ariccia, Italy; 5 Department of Molecular Medicine, University of Padova, 35121 Padova, Italy; camilla.onelli@studenti.unipd.it; 6 Department of Information Engineering, University of Padova, 35131 Padova, Italy; elena.pegolo@phd.unipd.it; 7 Fondazione Santa Lucia IRCCS, 00179 Rome, Italy

**Keywords:** hypomimia, Parkinson’s disease, facial expressions, amimia, emotion recognition, face, facial bradykinesia

## Abstract

Facial movements are crucial for social and emotional interaction and well-being. Reduced facial expressions (i.e., hypomimia) is a common feature in patients with Parkinson’s disease (PD) and previous studies linked this manifestation to both motor symptoms of the disease and altered emotion recognition and processing. Nevertheless, research on facial motor impairment in PD has been rather scarce and only a limited number of clinical evaluation tools are available, often suffering from poor validation processes and high inter- and intra-rater variability. In recent years, the availability of technology-enhanced quantification methods of facial movements, such as automated video analysis and machine learning application, led to increasing interest in studying hypomimia in PD. In this narrative review, we summarize the current knowledge on pathophysiological hypotheses at the basis of hypomimia in PD, with particular focus on the association between reduced facial expressions and emotional processing and analyze the current evaluation tools and management strategies for this symptom, as well as future research perspectives.

## 1. Introduction

Parkinson’s disease (PD) is the second most common neurodegenerative disorder of adult age [1], and is clinically characterized by bradykinesia, rigidity, and resting tremor [2]. Bradykinesia refers to the slowness of voluntary and involuntary movements, which is typically exacerbated during repetitive movements, with progressive reduction in speed and amplitude of motor act, defined as the “sequence effect” [3].

Hypomimia, often referred to as “masked face”, describes the reduction in facial movements and is a rather common feature of PD. The term “masked face” was first used by Charcot in 1860 [4], describing Parkinsonian facial expressions. In the following decades, the reduction in facial movements was included in the classical characteristics of Parkinsonian patients. Gowers, in his Manual of diseases of the nervous system, described the typical facial expression of Parkinsonian patients as “anxious and fixed, unchanged by any play of emotion” [5]. The term “amimia” was then used by Wilson [6], and “hypomimia” was introduced in the following decades. Hypomimia may have variable severity, from the reduction in blinking rate to fixed facial postures, leading to a “masked face” [1,2,7]. Despite the conspicuous literature on motor symptom impairment in PD, research on facial motor feature impairment has been rather scarce. The issue is, however, of particular interest, since physiological motor control of facial muscles is significantly different from limbs [8]. Moreover, facial movements have been linked with social and emotional behavior [9,10], and facial expressions have been associated with emotion recognition and processing in several conditions, such as depression [11,12,13] and cognitive impairment [13,14,15,16]. In PD, a variety of non-motor symptoms has been described, including cognitive impairment and mood disorders [17]; therefore, the impact of hypomimia on emotional processing and social behavior, beyond the simple masked face, may be even more relevant.

Clinical evaluation has long been the only tool to quantify the severity of hypomimia. In recent years, however, the advent of face recognition tools, computer-based image analysis, and machine learning paradigms led to increased interest toward facial expressions assessment and hypomimia in PD. In this paper, we review the current knowledge on pathophysiological hypotheses at the basis of hypomimia in PD, with a particular focus on the association between reduced facial expressions and emotional processing, and summarize current evaluation tools and management of this symptom, as well as future research perspectives.

## 2. Search Strategy

A search was performed in January 2023 on PubMed, Scopus, Embase, and PEDro databases for papers published in English, without publication date limit. Keywords related to (i) hypomimia (e.g., “hypomimia OR amimia OR (face bradykinesia) OR (facial expression*) OR (facial masking) OR (blink*)” for PubMed); (ii) Parkinson’s disease (e.g., “(parkinson’s disease) OR (parkinson) OR “Parkinson Disease” [Mesh])”, for PubMed) were used. Search fields were restricted to the abstract, title, and keywords. Included papers were original peer-reviewed scientific journal articles, editorials, case studies, letters, and reviews. Studies not examining hypomimia in the context of PD were excluded. Moreover, the search was enlarged to the references of the originally selected papers, and a manual search was conducted on IEEE Xplore to identify additional studies. The search was repeated in August 2023 to identify potential missing articles.

Two independent reviewers (EB and DR) screened the titles and abstracts of all studies to identify potentially relevant articles. Discrepancies in the evaluation were discussed with the remaining authors. Duplicates were manually removed. Full texts of all included studies were then obtained and reviewed.

The following five parameters were reviewed from the retrieved articles by all authors and checked by two independent reviewers (EB and DR): (1) study characteristics; (2) participant characteristics; (3) tools for measuring hypomimia; (4) outcome measures; (5) main findings. Quantitative analysis and assessment of the risk of bias were not performed due to the narrative design of the study.

## 3. Clinical Features and Pathophysiology of Hypomimia

### 3.1. Clinical Features of Hypomimia and Basics of Facial Motor Control

Hypomimia is defined as the reduction in or loss of spontaneous facial movements and emotional facial expression. This may vary in presentation and severity, and usually worsens over time along with PD progression, ranging from the reduction in blinking rate to fixed facial facies [18]. Hypomimia is often an underestimated symptom in PD patients, but the absent expressiveness can lead to relevant consequences on the patient’s life; in particular, it is associated with social deterioration and stigmatization [19,20]. Indeed, the more hypomimic an individual is, the less interest is shown by other subjects in interacting with them [21]. Even relatives of PD subjects are less inclined to interact with the patient the more he presents “facial masking” [20]. Hypomimia can be confused with negative feelings and become an obstacle to building inter-individual relationships, underlying interpersonal and psychological difficulties in Parkinsonian patients. Hypomimia can, therefore, have an adverse impact on close relationships and psychological well-being [22].

The Unified Parkinson Disease Rating Scale (UPDRS), the most widely used scale to globally assess the severity of PD symptoms [23,24], recognizes a clinical progression of hypomimia from reduced blinking rate to diminished lower face and perioral movement, such as spontaneous smiling, up to a global reduction in movements with parted lips. This condition could make PD patients take on a “masked” or “poker” face with wider palpebral fissures, flattened nasolabial folds, reduced wrinkles around the mouth, and involuntarily opened lips [18]. Although motor symptoms of PD usually display asymmetry, hypomimia is generally symmetrical, with rare descriptions of hemi-hypomimia, homolateral to the most affected body side, being rarely reported [25].

The pathophysiological basis of hypomimia is unclear, and a complex intertwining between networks and brain areas is likely to play a role in its genesis. Indeed, the facial motor system is extremely complex and significantly different from the one controlling limbs. In humans, different cortical areas have been associated with facial motor control, including the motor and premotor cortices, supplementary motor area [8], and a mesial “face motor area” [26]. A partial somatotopic face organization exists at different levels of the motor system, including motor cortices and basal nuclei [8,27,28]. However, the complexity of face motor control goes beyond the single muscle representation, and a mosaic of brain structures comes into play, probably as the consequence of the high symbolic content of facial movements [8]. Neurophysiological and clinical observation also support the concept of a different motor system organization for the upper and lower portions of the face. Clinical experience indicates that upper facial muscles are less involved in central facial palsies, probably due to the greater amount of bilateral innervation, a larger amount of subcorticobulbar input to these muscles, and the more extensive participation of areas other than motor cortices [7,8]. Transcranial magnetic stimulation studies indicate a role of the medial frontal cortex in upper facial muscle control, whereas M1 projections control mostly the contralateral lower facial muscles [29,30], even though evidence for M1 projections to the ipsilateral lower facial muscle in humans exists [31,32]. Furthermore, previous evidence supports the notion that different neural pathways, with a degree of overlapping, control involuntary and voluntary facial movements [7]. Indeed, voluntary facial paresis is frequently observed after lesions of motor areas but face emotional activation is often spared, most likely because projections from the mesial frontal areas are preserved [33,34]. In contrast, lesions involving the thalamus, the frontal and mesial temporal lobes, as well as operculum and insula result in emotional facial paresis [33,34]. Studies also suggested different brain involvement in spontaneous and posed emotional facial expressions. A network including the left temporo-occipito-parietal junction, left lateral prefrontal lobe, and basal temporal lobes seems to mediate spontaneous emotional facial expression [35,36]. Conversely, different areas, such as frontal lobe convexity and mesial frontal region, have been suggested to control posed expressions and voluntary face movements [29,30,32,37]. Eventually, the facial motor system exhibits peculiar features when compared to the limb motor system, such as the lack of muscle recurrent and reciprocal inhibition, the limited inertia during movement due to the minimal mass of the moved segments, the fact that facial muscles do not act on joints, and the reduced proprioceptive component, putatively vicariate by skin-sensitive afferents [7,8].

As mentioned above, facial muscles mediate a variety of involuntary and voluntary facial movements, ranging from stereotyped (e.g., blinking) to complex facies (e.g., grimace), with different neural bases underpinning them. Moreover, facial muscles have the peculiarity of generating prototypical emotional expressions (e.g., happiness, sadness, anger, etc.) as well as more complex and ambiguous ones, with an intrinsic emotional content. All these aspects may be affected by PD and will be addressed in the following sections.

### 3.2. Blinking and Voluntary Movements

Blinking rate is usually reduced from the early stage of PD [38,39,40,41]. This has been associated with dopaminergic denervation and is at least partially restored by dopamine replacement therapy [39]. However both cortical and subcortical structures are involved in spontaneous eye blinking in humans and animal models, including mesial frontal areas [42,43] and the spinal trigeminal complex [44]. The closing and opening phases of blinking are relatively preserved in people with PD, despite significantly longer inter-phase duration than healthy controls [38]. In addition, other studies reported substantially preserved blinking reflex in PD [45,46], thus suggesting the integrity of brainstem circuitry. Therefore, blinking rate alteration in PD is most likely attributable to changes in basal nuclei modulation, leading to reduced functional connectivity with mesial frontal region areas, including the supplementary motor areas, which subserve sequential movements, and to impaired switching between opening and closing eyelid phases [38]. This is also supported by the observation that spontaneous blinking rate, but not blinking reflex, is positively modulated in PD following deep brain stimulation (DBS) of the subthalamic nucleus [47].

Other observations showed that people with PD display difficulty in executing other non-emotional voluntary facial movements (orofacial movements, facial mimicry, etc.). These studies demonstrated slower movements in the perioral area during repetition of syllables both in terms of amplitude and velocity of movement, and suggested a relationship between abnormal facial movement and bradykinesia and rigidity [48,49,50]. This association has been investigated in other studies reporting correlations between hypomimia severity and bradykinesia, rigidity, and axial symptoms [51,52,53,54], suggesting a prominent role of dopaminergic system derangement in the genesis of hypomimia. Indeed, hypomimia severity has been recently associated with the level of nigrostriatal dopaminergic degeneration [55,56], and some studies reported the improvement of facial movement after L-DOPA administration [51,52,57,58]. Interestingly, hypomimia seems to improve during sleep [59]. This could possibly be due to descending motor pathway projections bypassing the extrapyramidal system [59].

Hypomimia could also point towards the identification of specific disease phenotypes. PD is characterized by alpha-synuclein aggregates, but it remains uncertain where the initial α-synuclein aggregates originate [60]. The theory that the disease may have a top-down (brain-first) phenotype and a bottom-up (body-first) phenotype is supported by several authors [61,62]. It was recently suggested that PD patients with rapid eye movement sleep behavior disorder (RBD) were compatible with a “body-first” trajectory, characterized by initial loss of cardiac 123I-metaiodobenzylguanidine (MIBG) signal and 11C-colonic donepezil signal, and only subsequently showed loss of putaminal 18F-dihydroxyphenylalanine (FDOPA) uptake. Conversely, patients without RBD showed a cerebral trajectory. Patients with the body-first phenotype typically exhibit RBD, and it can be associated with autonomic disturbances, constipation, and more rapid cognitive decline [61]. In the body-first phenotype, a more homogeneous degeneration of the nigrostriatal system is recognized, with initial enteric pathology, which spreads through vagal innervation, leading to more symmetric brainstem involvement [63]. It could be hypothesized that this could also be reflected in the fact that hypomimia is often symmetrical, regardless of the asymmetry of the motor symptoms. However, this feature could also be consistent with the rich bilateral involvement of cortical and subcortical areas and with the bilateral projections controlling face movements. Nevertheless, no studies specifically addressed the difference in hypomimia occurrence across identified phenotypes; therefore, no solid conclusion could be drawn. Research is dedicating a large effort to identify disease subtype specificities and clusters in PD; therefore, this aspect could be a highly relevant topic to investigate in future studies.

The evidence presented above highlights the complex phenomena underlying hypomimia and supports the notion that the pathophysiology of this symptom goes beyond a pure motor impairment. Another relevant aspect that has been investigated in the context of hypomimia is the interconnection between emotional processing and facial expressions. This will be discussed in the following section.

### 3.3. Emotional Processing and Facial Emotion Recognition

Facial emotion recognition is a critical aspect for appropriate social behavior and emotional functioning. Emotional processing allows communicating our needs and feelings and recognizing those of others and helps to predict our and others’ reactions and behaviors. Previous studies reported an overlap between areas activated during facial recognition and facial expression, including the premotor face area, the dorsal sector of pars opercularis of the inferior frontal gyrus, the superior temporal sulcus, the insula, and the amygdala [64], which plays a key role in orchestrating emotional outputs [65,66]. This complex network would act as an interface between the frontal component of the mirror neuron system and the limbic system, leading to the translation of expressions into its internally represented emotional meaning [64,67]. This concept is supported by the observation that limiting facial movements during facial emotion recognition reduces accuracy of performance [68]. Given this functional anatomic substrate, several studies focused on the association between reduced facial expressivity in people with PD and the altered facial emotion recognition, as well as the potential common neural mechanism between these two phenomena [10,67,69,70,71,72,73]. All but one small study [72] reported a variable degree of association between facial emotion recognition and decreased facial expression, supporting the association between these two features of the disease.

In people with PD, several domains of emotional processing are affected, such as prosody, faces, describing sensations, physiological arousal and feelings, expressing emotions, and emotional language [74]. However, it is not clear if PD selectively impairs the recognition of specific emotions or leads to an overall deficit. According to several authors, although all basic emotions are involved, the deficit of facial emotion recognition is greater for negative emotions [66,75]. This may pertain to a subcortical pathway that results in coarse but rapid visual information processing, involving the pulvinar, amygdala, and striatum [76,77]. This is a preserved route for its evolutionary relevance, because it allows one to recognize angry faces as a threatening stimulus through a preattentive and autonomic bias [78]. Although the hypothesis of the “angry faces advantage” has not been confirmed, it is interesting to note that previous studies reported a correlation between hypomimia severity and negative emotion recognition, particularly disgust [70,71]. However, other authors supported the hypothesis of the “happy faces advantage” and, to this end, a study from Argaud and colleagues [69] reported that, in people with PD, happiness is the most impaired facial expression and that they are less accurate in recognizing positive and neutral facial expressions. Nonetheless, it is important to consider that classifying a positive emotion without a proper context is much more difficult with respect to a negative one, and among the basic emotions, happiness is the only positive one.

Emotional disturbances have been traced to the reduced dopaminergic transmission both in nigrostriatal and mesocortical/mesolimbic pathways, as well as orbitofrontal and amygdalar areas. Temporal areas used for facial recognition are less active in people with PD [79]. In addition, basal nuclei are involved in emotional processing and cognitive functions as well [80]. The thalamocortical circuit passing through the basal nuclei may, indeed, modulate emotional processing by inhibiting irrelevant information (non-target emotions) and selecting those deemed relevant (target emotions) [80]. Progressive neuronal loss can make habitual processing more difficult, and thus complicate automatic control processes in people with PD [81]. In this respect, a study revealed that the reduction in putaminal dopamine transporter (DAT) is associated with lower activation of the ventrolateral prefrontal cortex in response to emotional gestures, and a greater number of errors in the recognition of these emotional gestures [82]. The basal nuclei dysfunction secondary to dopaminergic neuronal loss could represent a common pathological aspect with hypomimia. Indeed, previous evidence suggested that dopaminergic presynaptic deficit at the striatal level, as investigated by SPECT, correlates with the severity of facial mobility impairment [55,56]. The putative role of basal nuclei in both facial expressions and recognition have also been demonstrated in other disorders, such as Huntington’s disease, where both of these two aspects correlate with the degree of striatal atrophy [83].

Besides basal nuclei, morphometric analysis revealed decreased gray matter volume in numerous limbic, paralimbic, and neocortical associative temporo-occipital areas. Therefore, cortical atrophy could be associated with or exacerbate the disorder [84,85]. Interestingly, a study from Gasca-Salas and Urso [73] reported the association between cognitive impairment and hypomimia severity in a large cohort of PD patients. Similarly, Maycas-Cepeda and collaborators reported an association between cognitive status and hypomimia severity [53], thus supporting the hypothesis that decreased facial expression in PD may not be a purely motor and dopaminergic feature and might be related, at least partially, to higher brain functions [73]. Indeed, executive dysfunction also plays a role in facial emotion recognition. Working memory impairment can interfere with the ability to manage, operate, and memorize information, thus affecting facial emotional processing. In addition, in PD, there is a divided attention deficit, which makes it difficult to process different sources at the same time, and selective attention is also compromised [86].

Facial emotion recognition impairment may be more relevant in patients with left-dominant motor symptoms, considering the relatively greater role of the right hemisphere in processing feelings such as anger, fear, and sadness [87]. Some evidence suggests that facial emotion recognition deficit may be related to visuospatial disturbances in PD, again predominantly mediated by the right hemisphere [88,89]. The visual and emotional systems are closely connected, as demonstrated by the pathways between the amygdala and superior colliculus via the pulvinar, between the amygdala and orbitofrontal cortex, anterior cingulate cortex, and the visual regions in the temporal cortex [77]. Regarding the effect of laterality of symptoms on facial expressivity in PD patients, only one study investigated this aspect [90]. Interestingly, although left-sided and right-sided PD patients did not differ in overall amount of facial movement, the left-sided group was significantly slower in initiating two (anger and happiness) of the three emotions explored in the study, further supporting the notion that a greater right hemisphere involvement could lead to a higher degree of hypomimia [90].

Mood disorders are a further feature potentially influencing both emotional processing and facial expressivity. In depression, in fact, these two processes are commonly disrupted [11,91], and an association between reduced facial expressions and impaired facial emotion recognition has been described [11,12,13]. Mood disorders, including depression, apathy, and anxiety, are common non-motor features of PD, often preceding motor symptom onset [17]; therefore, this might contribute to the alteration of both facial expressions and emotional processing. Indeed, a previous study reported worse apathy in patients with hypomimia compared with those without [52]. Similarly, another recent study from Sampedro and collaborators [92] reported an association between the severity of apathy and hypomimia, as measured by the UPDRS in early PD patients. Interestingly, in this latter study, authors reported that hypomimia score was negatively associated with cortical thickness in multiple fronto-temporo-parietal regions involved in the decoding, recognition, and production of facial expression of emotions [92]. However, other studies did not find an association between depression and hypomimia severity [53] or differences in depression and anxiety between PD patients with and without hypomimia [52].

In addition, despite the impairment in making spontaneous expressions, in early studies, PD patients did not display a significant deficit in emotional mimicking [93]. This supports the idea of partial functional distinction between emotional and non-emotional facial movements and the greater involvement of the former in PD. Subsequent studies, however, led to inconsistent results, with some studies reporting normal posed facial expressions in PD patients [94], whereas disruption of posed expression has also been reported [95,96]. Also, PD patients displayed difficulties in facial mimicry [97,98] and in making incongruent expressions (e.g., to show anger while watching an amusing video) [96]. This alteration has been traced back to pathological changes occurring at the level of the amygdala [99].

Finally, it must be considered that no study on hypomimia in PD took into consideration the cultural influence on facial emotion recognition and processing. Indeed, previous studies highlighted the cultural commonalities and specificities of facial expressions of emotion [100]. This aspect could be investigated in future studies on hypomimia in PD patients.

In conclusion, most evidence supports the association between facial emotion recognition and hypomimia in PD patients. The interplay between these two features has not yet been fully clarified and probably involves several networks and brain areas at cortical and subcortical levels. Understanding such associations is highly relevant, since one may hypothesize that treating one aspect could affect the other one. Indeed, preliminary results seem to support this idea by showing that facial mimicry exhibits a positive effect on emotional processing in PD patients [69].

## 4. Assessment of Hypomimia

### 4.1. Clinical Evaluation

Clinical evaluation of hypomimia is rather challenging, and various approaches have been applied to assess the presence and severity of the symptom. The Unified Parkinson’s Disease Rating Scale (UPDRS) has been the most used tool for quantifying hypomimia in PD. This is the most frequently utilized rating scale to globally assess the severity of Parkinsonism, originally developed in 1987 (UPDRS) [24] and revisited in 2008 by the International Movement Disorders Society (MDS-UPDRS) [23]. In the original version [24], item 19 explored facial expression by rating it with a score from 0 (“normal”) to 4 (“masked or fixed facies, with severe or complete loss of facial expression; lips parted 1/4 inch or more”), taking into account the reduction in expressivity as judged by the assessor and the parting of the lips. In the MDS-UPDRS, item 3.2 refers to facial expression, instructing the rater to consider the eye-blink frequency, masked facies or loss of facial expression, spontaneous smiling, and the parting of lips [23]. MDS-UPDRS scores facial expression from 0 (Normal: Normal facial expression) to 4 (Severe: Masked facies with lips parted most of the time when the mouth is at rest).

In most published studies, hypomimia in PD patients was evaluated through the UPDRS or the MDS-UPDRS [52,53,55,56,58,71,73,97,101,102,103,104,105]. However, it must be noted that such assessment suffers from subjective evaluation by the assessor and from the bias of high inter- and intra-rater variability. Moreover, the limited five-point scale may not be sensitive enough to detect subtle changes in patients’ conditions. Eventually, no study investigated the responsiveness of the score to changes to treatment and interventions. The scoring system of UPDRS and MDS-UPDRS for hypomimia is shown in Table 1.

Other studies applied different approaches to quantify hypomimia, such as questionnaires originally developed to assess emotional expressivity (i.e., Ekman test, New York Emotion Battery, Berkeley Expressivity Questionnaire, Upper and Lower Face Apraxia Test) [67,89,106,107], or subjective quantitative and/or qualitative evaluation from clinical experts [10,25,50,108,109]. These latter approaches, however, suffer from limitations similar to the UPDRS in terms of inter- and intra-rater variability and have not been fully validated for the specific aim of evaluating hypomimia in PD.

### 4.2. Instrumental Evaluation

Given the limitations of the clinical methods to evaluate hypomimia in PD, attempts have been made to develop different instrumental and objective techniques. The first approach to quantify differences in face mobility between PD patients and healthy controls (HC) was based on electromyography (EMG). Back in 1982, Hunker and collaborators [50] reported differences in EMG activation of diverse facial muscles between the two populations. EMG has been used more recently for measuring spontaneous and voluntary expressions [98] and facial mimicry in reaction to different stimuli [69,97]. One study integrated the use of EMG with electro-oculography to study eye movement in relation to face muscles [110]. Wu and colleagues [111], in their preliminary work, focused on the integration of EMG with both electrocardiogram and face video recording with the final aim of automatically recognizing the activation of Action Units (AUs). AUs represent the fundamental actions of facial expressions and are defined as the contraction of either single muscles or a combination of them along with different eye and mouth movements. They were defined by Ekman and Friesen in their Facial Action Coding System (FACS) and are still widely used for facial analysis applications [112].

Despite the well-established use of motion analysis for studying motor impairments in PD, only a few studies performed by a single research group employed this technology to assess hypomimia in PD. In particular, these authors compared facial kinematic variables (i.e., velocity and amplitude) in PD and HC subjects via 3D optoelectronics cameras applying reflective markers on the face [54,72].

Given the complex experimental setup of the previously described approaches, however, extensive effort has focused on evaluating hypomimia via video assessment in different forms, considering its straightforward usability. A line of research aimed at distinguishing the PD population from HC with machine or deep learning techniques. The typical pipeline consists of extracting facial features from large databases of videos/images and then testing different models to classify the subjects’ physiological or pathological status. Several studies employed videos/images of smiles as input for the analysis [113,114,115], whereas some of them did not specify the expression(s) under analysis [103,116]. Gomez-Gomez and colleagues [117], in their work, enriched the detection of PD from single images or sequences of images with recently developed affective models and employed domain adaptation to existing models for face recognition and AU detection. On another level, different approaches have been tested to quantify face mobility from videos/images. The final aim of these methods is either to highlight impairments in the PD population with respect to HC or, more recently, to develop quantitative indexes of hypomimia. Katsikitis and Pilowski [118,119] first tackled the problem in PD and subsequently in depression by computing facial measures from images of smiles extracted from videos. PD subjects showed differences from HC in the frequency of smiling and in the degree of mouth opening. As already reported in some studies, FACS has been employed to quantify differences in facial expression between PD and HC, both with trained coders’ ratings [120] and with automatic software for AU detection (OpenFace version 2.0) [121,122].

Other works developed metrics to quantify the degree of impairment. Bowers and colleagues developed an index based on the entropy of sequential images converted in grey scale [123], which was used by other authors as well [90]. More recently, the former group applied the same methodology to quantify the effects of levodopa on facial expressions [58]. Eventually, other authors used the extraction of geometric features from images of emotions in PD to quantify hypomimia [124] and create new indexes of impairment [125] also from images captured via smartphones [104].

Most of these studies employed videos collected via commercial cameras either in the clinical setting or at home. Subjects were usually sitting with the camera placed at eye level and the examiner asked them to perform a given task (i.e., emotions or expressions) or specific emotion-eliciting stimuli were displayed through the screen itself. Analysis of the videos was performed mainly offline with ad hoc codes that typically involve adapted pipelines employed in the face emotion recognition systems, as mentioned above. Indeed, it is essential to highlight that these tools pertain primarily to research studies on hypomimia, and they still need to be integrated into clinical practice.

In summary, quantifying hypomimia severity has been addressed through different approaches, with video analysis being the most explored. However, some issues still remain open. As anticipated above, it is crucial to highlight that the methodologies presented here refer to a great variety of expressions and emotions, from the analysis of the smile only to all the basic emotions (as defined by Darwin in 1872 [126]). In addition, the assessment of emotions is tricky, not only in a pathological population, due to the intrinsic subjectivity present. Different approaches have been employed to study them. Some studies elicited the emotions under analysis with the support of audio or videos (spontaneous facial expressions), whereas in other reports, subjects were asked to intentionally display the required emotion (posed facial expressions). Finally, acquisitions and elaborations of either videos or images may vary greatly across different studies. For all these reasons, there is a need to standardize the instrumental evaluation of hypomimia in order to provide easy-to-use and accessible tools for clinicians that could also be employed to assess rehabilitation treatments.

## 5. Management of Hypomimia

### 5.1. Pharmacological Treatments

Even the most recent recommendations from the MDS for the management of motor symptoms in PD patients [127] do not provide indications regarding hypomimia. This is mainly due to the lack of studies comprehensively investigating pharmacological and non-pharmacological management of this symptom and the lack of standardization and definite reliability of the assessment. As discussed previously, the mechanisms underpinning hypomimia are rather complex and involve several networks and brain areas. However, previous studies reported the association between the severity of hypomimia and other motor symptoms, such as bradykinesia and rigidity [51,52,53,54]. Moreover, the severity of hypomimia correlated with the level of nigrostriatal dopaminergic degeneration, suggesting a pathogenetic role [55,56]. Taken together, these observations provided the rationale for dopaminergic treatment of hypomimia in PD patients. Only a few studies explored this aspect; hence, levodopa demonstrated some efficacy in ameliorating facial mobility [51,52,57,58]. Thus, PD patients displayed more marked reduction in pain expression, as assessed through videotapes during pain stimulation (Priebe et al., 2015), and more marked perioral stiffness [51] in OFF compared with ON state. In one study from Ricciardi and colleagues [52], 89 PD patients were evaluated by expert clinicians in ON and OFF pharmacological states, hypomimia being quantified by means of the MDS-UPDRS. Under these conditions, hypomimia scores were significantly lower in ON condition than in OFF. Finally, a recent study testing dynamic facial expression, measured through an entropy video analysis of facial movement, showed greater facial movements in ON compared with OFF condition [58]. All these findings suggest a potential beneficial effect of dopaminergic treatment on hypomimia in PD patients. However, most of these studies included small cohorts, used non-standardized assessment methods, and did not include a blinded evaluation of patients. More solid studies on large cohorts, applying a blinded design and standardized outcome measures, should be encouraged to evaluate the effect of dopamine replacement therapy on hypomimia in PD patients.

### 5.2. Non-Pharmacological Treatments

Despite the severe negative impact of hypomimia on the quality of life and social wellness of patients and the potential rationale for effectiveness, to date, only very few studies have investigated the effectiveness of rehabilitation strategies on hypomimia in PD. These mainly focused on orofacial physiotherapy [102,128] and speech therapy [106,109]. Regarding physiotherapy, Katsikitis and collaborators [128] conducted a controlled study in 16 PD patients. The protocol consisted of orofacial physiotherapy treatment and included, among other things, brushing muscles, applying ice to them, or asking subjects to blow through a straw to stimulate facial muscles. Treatment was performed for 1 h twice a week. Hypomimia was evaluated using a microcomputer-based analysis on videotapes recorded during natural conversation. Subjects in the experimental group achieved significant improvements in some facial mobility measures (e.g., mouth opening measure, mid-top-lip measures, etc.).

Conversely, in the work by Ricciardi and colleagues [102], two different physiotherapy approaches were tested in 40 PD patients. In the first group, patients underwent standard facial rehabilitation with facial muscle strengthening exercises delivered through a DVD guide, whereas in the second one, patients received facial rehabilitation through a proprioceptive/recognition approach. The effects of treatments were evaluated by means of the MDS-UPDRS and with a computerized analysis of facial expression (E-Motion). Clinical scores improved in the proprioceptive/recognition group but not in the standard treatment group. The authors explained the results as an effect of the proprioceptive emotional processing component that is present in the proprioceptive/recognition approach and would act on networks and processes that are altered in PD patients.

Besides physiotherapy, a few studies focused on speech therapy, in particular Lee Silverman Voice Treatment (LSVT), in PD patients with hypomimia. In a work by Spielman and colleagues [109], LSVT was compared with respiratory therapy in 44 PD patients, finding greater improvement in facial mobility in the former group, as evaluated by expert raters. Authors hypothesized that through high-stress phonatory exercises, which involve a wider opening of the mouth and prolonged tension of the facial muscles, the entire seventh cranial nerve is stimulated, inducing an increase in movement. Following these hypotheses, Dumer and collaborators compared LSVT with articulatory orofacial treatment (ARTIC) in PD patients with hypomimia [106]. Hypomimia was evaluated from videotapes by raters using FACS. The authors reported an improvement in facial expressivity in the LSVT group compared with ARTIC. Taken together, these studies suggest a potential benefit of physiotherapy and LSVT on hypomimia in PD patients. However, given the marked heterogeneity in protocols and outcome measures and the small cohorts involved, further studies are necessary to draw firm conclusions.

Regarding other non-pharmacological approaches, only one case series investigated the effect of non-invasive brain stimulation on hypomimia [129]. In this work, authors reported that picoTesla range magnetic fields improved hypomimia in four patients with PD. However, this anecdotal evidence has not been confirmed by other studies, and no reports are available, to the best of our knowledge, on the possible effect of non-invasive brain stimulation on hypomimia in PD patients.

## 6. Conclusions and Future Directions

Studies on hypomimia in PD patients suggest that a role of dopaminergic dysfunction and an association with motor symptom severity is present. However, the underlying process seems to go beyond a pure motor impairment, and an association between facial emotion recognition and altered facial expression is observed, as well as a role of non-motor manifestations such as mood disorders and cognitive impairment. The interplay between these aspects is not fully clarified and is likely to involve several networks and brain areas at cortical and subcortical levels. In this framework, we suggest that hypomimia is considered to be the result of variably combined motor and non-motor features, and future studies investigating this manifestation in people with PD should include both motor and non-motor evaluation.

The lack of valid and reliable instruments to quantify hypomimia represents one of the major limitations in this research area. Moreover, most of the clinical evaluation tools suffer from high inter- and intra-rater variability, and no study has investigated their responsiveness to pharmacological and non-pharmacological interventions. These issues have been addressed recently by several technology-enhanced approaches, in particular automated video analysis and machine learning application. However, studies used different quantification methods and evaluations, as well as sparse facial expression-eliciting protocols. Therefore, the need to standardize the instrumental hypomimia assessment in order to provide an easy-to-use and accessible tool for clinicians is crucial and should be a primary goal of future research focusing on technology application to facial mobility in PD patients.

Finally, another prominent aspect emerging from our review is the lack of studies investigating the management of hypomimia through pharmacological and non-pharmacological approaches. Pathophysiological and preliminary clinical studies suggested a rationale for dopaminergic replacement therapy and facial rehabilitation efficacy; nevertheless, studies are scarce and most evidence is low-level due to the small number of enrolled patients and the heterogeneity of evaluation and treatment protocols. More studies on large cohorts, applying a blinded design and standardized, objective outcome measures, must be encouraged to address this unmet need in the management of people with PD.

## Figures and Tables

**Table 1:** Comparison of scoring system for hypomimia severity of item 19 of UPDRS [24] and item 3.2 of MDS-UPDRS [23].

Score	UPDRS	MDS-UPDRS
0	Normal.	Normal: Normal facial expression.
1	Minimal hypomimia, could be normal “Poker Face”.	Slight: Minimal masked facies manifested only by decreased frequency of blinking.
2	Slight but definitely abnormal diminution of facial expression.	Mild: In addition to decreased eye-blink frequency, masked facies present in the lower face as well, namely fewer movements around the mouth, such as less spontaneous smiling, but lips not parted.
3	Moderate hypomimia; lips parted some of the time.	Moderate: Masked facies with lips parted some of the time when the mouth is at rest.
4	Masked or fixed facies with severe or complete loss of facial expression; lips parted 1/4 inch or more.	Severe: Masked facies with lips parted most of the time when the mouth is at rest.

## Data Availability

No new data were created or analyzed in this study. Data sharing is not applicable to this article.
